# Decreased CO_2_ Levels as Indicators of Possible Mechanical Ventilation-Induced Hyperventilation in COVID-19 Patients: A Retrospective Analysis

**DOI:** 10.3389/fpubh.2020.596168

**Published:** 2021-01-08

**Authors:** Di Hu, Jinpeng Li, Rongfen Gao, Shipei Wang, Qianqian Li, Sichao Chen, Jianglong Huang, Yihui Huang, Man Li, Wei Long, Zeming Liu, Liang Guo, Xiaohui Wu

**Affiliations:** ^1^Department of Plastic Surgery, Zhongnan Hospital of Wuhan University, Wuhan, China; ^2^Department of Thyroid and Breast Surgery, Zhongnan Hospital of Wuhan University, Wuhan, China; ^3^Department of Rheumatology and Immunology, Tongji Medical College, Tongji Hospital, Huazhong University of Science and Technology, Wuhan, China; ^4^Department of Neurosurgery, Zhongnan Hospital of Wuhan University, Wuhan, China

**Keywords:** infectious disease, pneumonia, COVID-19, CO_2_, mechanical ventilation

## Abstract

**Background:** Six months since the outbreak of coronavirus disease (COVID-19), the pandemic continues to grow worldwide, although the outbreak in Wuhan, the worst-hit area, has been controlled. Thus, based on the clinical experience in Wuhan, we hypothesized that there is a relationship between the patient's CO_2_ levels and prognosis.

**Methods:** COVID-19 patients' information was retrospectively collected from medical records at the Leishenshan Hospital, Wuhan. Logistic and Cox regression analyses were conducted to determine the correlation between decreased CO_2_ levels and disease severity or mortality risk. The Kaplan-Meier curve analysis was coupled with the log-rank test to understand COVID-19 progression in patients with decreased CO_2_ levels. Curve fitting was used to confirm the correlation between computed tomography scores and CO_2_ levels.

**Results:** Cox regression analysis showed that the mortality risk of COVID-19 patients correlated with decreased CO_2_ levels. The adjusted hazard ratios for decreased CO_2_ levels in COVID-19 patients were 8.710 [95% confidence interval (CI): 2.773–27.365, *P* < 0.001], and 4.754 (95% CI: 1.380–16.370, *P* = 0.013). The adjusted odds ratio was 0.950 (95% CI: 0.431–2.094, *P* = 0.900). The Kaplan-Meier survival curves demonstrated that patients with decreased CO_2_ levels had a higher risk of mortality.

**Conclusions:** Decreased CO_2_ levels increased the mortality risk of COVID-19 patients, which might be caused by hyperventilation during mechanical ventilation. This finding provides important insights for clinical treatment recommendations.

## Background

In December 2019, an outbreak of pneumonia of unknown etiology was reported in Wuhan, China, which then rapidly evolved into a pandemic (1). By January 7, 2020, Chinese scientists had rapidly isolated the novel coronavirus, the severe acute respiratory syndrome coronavirus 2 (SARS-CoV-2), with an incubation period of 2–14 days, and a potential asymptomatic human-to-human transmission; it is known to cause the coronavirus disease (COVID-19) (2–4). COVID-19 has been controlled in China, although the global number of infections continues to grow rapidly and has led to more than five million infections and 630,000 deaths (5).

In COVID-19 patients, fever and cough are the most common symptoms. There may also be uncommon symptoms, such as diarrhea (6). Thus, researchers have found that SARS-CoV-2 affects multiple organs in addition to the patients' lungs, based on the understanding garnered from COVID-19 studies. This explains the pathological changes identified from the minimal autopsies of three patients who died of COVID-19 in Chongqin, China (7–9). Studies have shown that the main targeted organs of SARS-CoV-2 are the lungs and airways. Furthermore, damage to other organs significantly increases the mortality rate of COVID-19 patients (10).

The measurement of carbon dioxide (CO_2_) level in blood is vital not only for the early detection of respiratory depression and airway disorders but also for airway management (11). Hypoxemia and hypercapnia predicted poor prognosis for COVID-19 patients in a previous study (4). Hence, this study aimed to investigate whether decreased CO_2_ levels would influence the prognosis of COVID-19 patients.

## Methods

### Study Design and Participants

In this retrospective study, we collected data from 1,880 patients, who were clinically diagnosed with COVID-19 between February 8, 2020, and March 19, 2020, at Wuhan Leishenshan Hospital. Exclusion criteria included missing data on mortality and CO_2_ level, pregnancy, death on admission, embolization, and transfer to any other hospital; thus, 1,776 patients were included finally. Data about demographics, medical history, treatment, laboratory findings, and imaging data were collected from the patients' original medical records. Two physicians independently reviewed these data.

This study was approved by the Research Ethics Commission of the Zhongnan Hospital of Wuhan University (approval number: 2020074). The need for patient consent was waived by the ethics committee because of the urgent need for insights into this rapidly evolving infectious disease.

### Primary Outcomes in This Study

In this study, the survival and illness severity of COVID-19 patients during hospitalization and images obtained from computed tomography (CT) scan were used to evaluate the patients' primary outcomes. However, survival was the most significant indicator. According to the Seventh Interim Guidance of Diagnosis and Treatment of COVID-19 published by the Chinese National Health Commission, one patient was staged into mild COVID-19 in this study. Thus, the severity of COVID-19 was categorized into three degrees: mild/common, severe, and critical.

Furthermore, after fulfilling the common standard criteria, all chest CT images were inspected and independently categorized by two experienced radiologists using the following scoring system according to previous studies and the characteristics of COVID-19. Score 1 included ground-glass opacities (GGO) characteristics, reticulation or cord change, consolidation, and pleural effusion, in which each feature was assigned one point, and Score 1 was the sum of these features. Score 2 (from 0 to 4 points) was generated depending on the area of involvement of the lung lobes as follows: no involvement, 0; < 25% involvement, 1; 26–50% involvement, 2; 51–75% involvement, 3; 76–100% involvement, 4; the total score was the sum of scores 1 and 2.

### Statistical Analyses

Continuous variables with normal distribution are presented as mean ± standard deviation (SD) or median and interquartile range (IQR). A CO_2_ level ≤23 mmol/L was considered a decreased level (normal CO_2_ range: 23–31 mmol/L). Furthermore, differences in continuous variables between the groups (decreased and non-decreased levels of CO_2_), were determined using independent group *t*-test or the Mann-Whitney *U*-test. Categorical variables are presented as frequencies and percentages. For the proportions of categorical variables, the chi-square test was used to compare participants with decreased and non-decreased CO_2_ levels. When parameters were expected to have a count ≤5, the Fisher exact test was used.

To determine whether the decreased CO_2_ levels would influence the prognosis of COVID-19 patients, we used Cox regression analysis, after adjusting for age, history of cardiovascular disease, erythrocyte count, hemoglobin, leucocyte count, platelet count, lymphocyte count, and oxygen support. Furthermore, Kaplan-Meier analyses with log-rank tests were used to analyze the survival trends of patients.

All statistical analyses were performed using SPSS (version 23.0 for Windows) and EmpowerStats (version 2.0). A two-sided *P* ≤ 0.05 was considered statistically significant.

## Results

### Demographics, Clinical Information, and Laboratory Findings

The demographic characteristics and symptoms of this study cohort of 1,776 patients are presented in Table 1. The ratio of female to male patients was approximately one. The IQR value of age in this study population was 59 (48–68) years, with no apparent differences in the groups with decreased and non-decreased CO_2_ levels.

**Table 1:** Demographic characteristics and symptoms of 1,776 patients with COVID-19.

**Covariates**	**Levels**	**All patients (*n* = 1,776), *n* (%)**	**Non-declined CO_**2**_ (*n* = 1,343), *n* (%)**	**Declined CO_**2**_ (*n* = 433), *n* (%)**	***P*-value**
Gender					0.800
	Female	934 (52.59)	704 (75.37)	230 (24.63)	
	Male	842 (47.41)	639 (75.89)	203 (24.11)	
Age, median (IQR)		59 (48–68)	59 (49–68)	58 (47–67)	<0.001
Any comorbidity					
	Cardiovascular diseases	352 (19.82)	249 (70.74)	103 (29.26)	0.017
	Pulmonary diseases	89 (5.01)	62 (69.66)	27 (30.34)	0.179
	Endocrine diseases	135 (7.60)	104 (77.04)	31 (22.96)	0.690
	Malignancy	64 (3.60)	46 (71.88)	18 (28.13)	0.477
	Digest system diseases	45 (2.53)	35 (77.78)	10 (22.22)	0.733
	Neurological diseases	55 (3.10)	39 (70.91)	16 (20.09)	0.409
Initial symptoms, *n* (%)					
	Fever or fatigue	615 (34.50)	456 (74.10)	274 (34.60)	0.293
	Respiratory symptoms	626 (35.25)	465 (74.28)	161 (25.72)	0.333
	Digestive symptoms	82 (4.62)	52 (63.41)	30 (36.59)	0.008
	Neurological symptoms	26 (1.46)	20 (76.92)	6 (23.08)	0.876
	Other	26 (1.46)	19 (73.08)	7 (26.92)	0.761

In patients with cardiovascular comorbidity, a significant difference was observed between decreased and non-decreased CO_2_ levels. However, there were no significant differences in other comorbidities, including pulmonary disease, endocrine disease, malignancy, and neurological disorders. Furthermore, among COVID-19 patients with decreased or non-decreased CO_2_ levels, those with gastrointestinal disorders showed a significant difference. However, concerning fever, fatigue, or respiratory and neurological symptoms, there were no significant intergroup differences (Table 1).

We analyzed the laboratory results and the blood coagulation tests of patients in two groups (Table 2), and most of the laboratory indicators showed significant differences. The results of the blood coagulation test, except fibrinogen and thrombin time, showed significant intergroup differences among COVID-19 patients. The clinical treatment and outcomes are presented in Table 2. Anticoagulants and types of oxygen support significantly differed among patients in the two groups. However, the use of antiviral drugs, corticosteroids, and traditional Chinese medicine showed no significant differences between the groups. Concerning outcomes, disease progression showed a significant difference, with no significant difference in other outcome parameters.

**Table 2:** Laboratory and blood coagulation test results, clinical treatment, and outcomes of 1,776 patients with COVID-19.

**Covariate**	**All patients (*n* = 1,776), Median (IQR)/*n* (%)**	**Non-declined CO_**2**_ (*n* = 1,343), Median (IQR)/*n* (%)**	**Declined CO_**2**_ (*n* = 433), Median (IQR)/*n* (%)**	***P*-value**
Laboratory test				
Leucocyte count, × 10^9^/L				0.035
3.5–9.5	1,585 (89.35)	1,211 (76.40)	374 (23.60)	
<3.5	104 (5.86)	75 (72.12)	29 (27.88)	
>9.5	85 (4.79)	55 (64.71)	30 (35.29)	
Neutrophil count, × 10^9^/L				0.029
1.8–6.3	1,553 (87.54)	1,185 (76.30)	368 (23.70)	
<1.8	116 (6.54)	88 (75.86)	28 (24.14)	
>6.3	105 (5.92)	68 (64.76)	37 (35.24)	
Lymphocyte count, × 10^9^/L				0.848
1.1–3.2	1,457 (82.13)	1,103 (75.70)	354 (24.30)	
<1.1	291 (16.40)	216 (74.23)	75 (25.77)	
>3.2	26 (1.47)	22 (84.62)	4 (15.38)	
Erythrocyte count, × 10^12^/L				0.820
4.3–5.8	636 (35.85)	477 (75.00)	159 (25.00)	
<4.3	1,127 (63.53)	855 (75.87)	272 (24.13)	
>5.8	11 (0.63)	9 (81.82)	2 (18.18)	
Monocyte count, × 10^9^/L				0.012
0.1–0.6	1,251 (70.52)	968 (77.38)	283 (22.62)	
<0.1	6 (0.34)	3 (50.00)	3 (50.00)	
>0.6	517 (29.14)	370 (71.57)	147 (28.43)	
Hemoglobin, g/L				0.664
130.0–175.0	712 (40.14)	535 (75.14)	177 (24.86)	
<130.0	1,057 (59.58)	803 (75.97)	254 (24.03)	
>175.0	5 (0.28)	3 (60.00)	2 (40.00)	
Platelet count, × 10^9^/L				0.011
125.0–350.0	1,546 (87.15)	1,185 (76.65)	361 (23.35)	
<125.0	76 (4.28)	48 (63.16)	28 (36.84)	
>350.0	152 (8.57)	108 (71.05)	44 (28.95)	
Albumin, g/L				0.921
40–55	449 (25.35)	340 (75.72)	109 (24.28)	
<40	1,322 (74.65)	998 (75.49)	324 (24.51)	
Alanine aminotransferase, U/L				0.918
9–50	1,421 (80.24)	1,076 (75.72)	345 (24.28)	
<9	96 (5.42)	71 (73.96)	25 (26.04)	
>50	254 (14.34)	191 (75.20)	63 (24.80)	
Aspartate aminotransferase, U/L				0.175
15–40	1,304 (73.63)	991 (76.00)	313 (24.00)	
<15	317 (17.90)	243 (76.66)	74 (23.34)	
>40	150 (8.47)	104 (69.33)	46 (30.67)	
Total bilirubin, μmol/L				0.099
5.0–21.0	1,582 (89.33)	1,207 (76.30)	375 (23.70)	
<5.0	120 (6.78)	82 (68.33)	38 (31.67)	
>21.0	69 (3.90)	49 (71.01)	20 (28.99)	
Creatinine, μmol/L				<0.001
64.0–104.0	812 (45.72)	627 (77.22)	185 (22.78)	
<64.0	877 (49.38)	674 (76.85)	203 (23.15)	
>104.0	87 (4.90)	42 (48.82)	45 (51.72)	
Procalcitonin, ng/mL				0.002
<0.05	999 (66.42)	770 (77.08)	229 (22.92)	
> =0.05	505 (33.58)	352 (69.70)	153 (30.30)	
Interleukin-6, pg/mL				0.247
0–7.0	602 (83.96)	445 (73.92)	157 (26.08)	
>7.0	115 (16.04)	79 (68.70)	36 (31.30)	
SARS-CoV-19 IgM				0.598
No	387 (64.61)	303 (78.29)	84 (21.71)	
Yes	212 (35.39)	162 (76.42)	50 (23.58)	
SARS-CoV-19 IgG				0.772
No	49 (8.67)	37 (75.51)	12 (24.49)	
Yes	516 (91.33)	399 (77.33)	117 (22.67)	
Blood coagulation test				
Prothrombin time, s				<0.001
9.4–12.5	1,461 (92.41)	1,126 (77.07)	335 (22.93)	
<9.4	1 (0.06)	1 (100.00)	0 (0)	
>12.5	119 (7.53)	64 (53.78)	55 (46.22)	
International Normalized Ratio				0.004
0.8–1.3	1,504 (85.13)	1,144 (76.06)	360 (23.94)	
<0.8	19 (1.20)	14 (73.68)	5 (26.32)	
>1.3	58 (3.67)	33 (56.90)	25 (43.10)	
Activated partial thromboplastin time, s				0.012
25.1–36.5	1,038 (65.65)	785 (75.63)	253 (24.37)	
<25.1	462 (29.22)	356 (77.06)	106 (22.94)	
>36.5	81 (5.12)	50 (61.73)	31 (38.27)	
Fibrinogen, (g/L)				0.291
2.38–4.98	1,178 (74.51)	883 (74.96)	295 (25.04)	
<2.38	307 (19.42)	240 (78.18)	67 (21.82)	
>4.98	96 (6.07)	68 (70.83)	28 (29.17)	
Thrombin time, s				0.930
< =16.6	237 (14.99)	178 (75.11)	59 (24.89)	
>16.6	1,344 (85.01)	1,013 (75.37)	331 (24.63)	
D-dimer, g/L	0.38 (0.21–0.89)	0.37 (0.20–0.86)	0.41 (0.23–1.05)	<0.001
Clinical treatment				
Drugs				
Antibiotic	515 (29.00)	377 (73.20)	138 (26.80)	0.130
Antiviral drugs	858 (48.31)	655 (76.34)	203 (23.66)	0.494
Antimalarial drugs	139 (7.83)	108 (77.70)	31 (22.30)	0.552
Anticoagulants	119 (6.70)	79 (66.39)	40 (33.61)	0.015
Corticosteroid	104 (5.86)	72 (69.23)	32 (30.77)	0.118
Vitamin C	246 (13.85)	187 (76.02)	59 (23.98)	0.876
Traditional Chinese medicine	1,523 (85.75)	1,159 (76.10)	364 (23.90)	0.247
Oxygen support				
Low–flow nasal cannula	269 (15.15)	226 (84.01)	43 (15.99)	<0.001
Positive pressure nasal cannula	34 (1.91)	27 (79.41)	7 (20.59)	0.603
High-flow nasal cannula	16 (0.90)	13 (81.25)	3 (18.75)	0.598
Invasive mechanical ventilation	5 (0.30)	1 (20.00)	4 (80.00)	0.004
ECMO	1 (0.06)	0 (0)	1 (100.00)	0.078
Outcomes				
CT scores				0.416
1–4	74 (39.57)	57 (77.03)	17 (22.97)	
5–7	113 (60.43)	81 (71.68)	32 (28.32)	
Disease progression				<0.001
Stableness/hospitalization	1 (0.06)	1 (100.00)	0 (0)	
Improvement/recover	1,738 (99.09)	1,323 (76.12)	415 (23.88)	
Death	15 (0.86)	4 (26.67)	11 (73.33)	
Days in hospital, median (IQR)	18 (13–24)	18 (13–24)	18 (12–23)	<0.001
ICU care	29 (90.63)	18 (62.07)	11 (37.93)	0.188
Severity on admission				0.359
Mild/common	1,473 (82.94)	1,123 (76.24)	350 (23.76)	
Severe	281 (15.82)	205 (72.95)	76 (27.05)	
Critical	22 (1.24)	15 (68.18)	7 (31.82)	
Severity at worst				0.226
Mild/common	928 (52.40)	718 (77.40)	210 (22.60)	
Severe	800 (45.20)	592 (74.00)	208 (26.00)	
Critical	43 (2.40)	31 (72.10)	12 (27.90)	

### Analysis of Patient Prognosis

**Figure 1:**
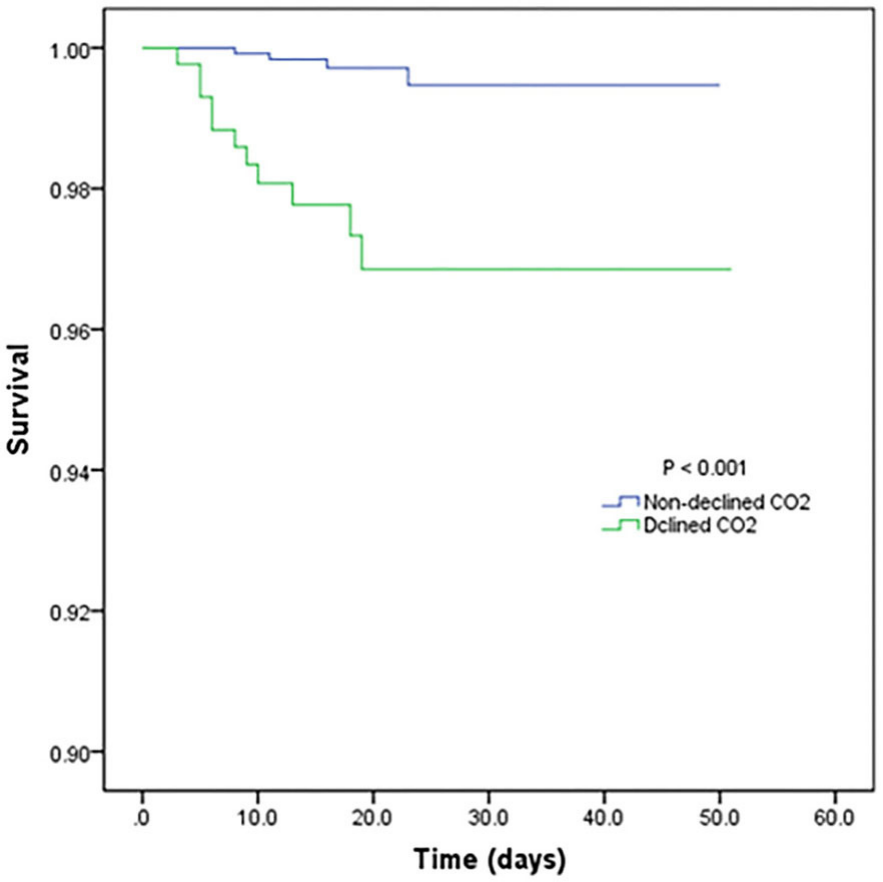
Kaplan-Meier survival curves for patients with declined and non-declined levels of CO_2_.

**Figure 2:**
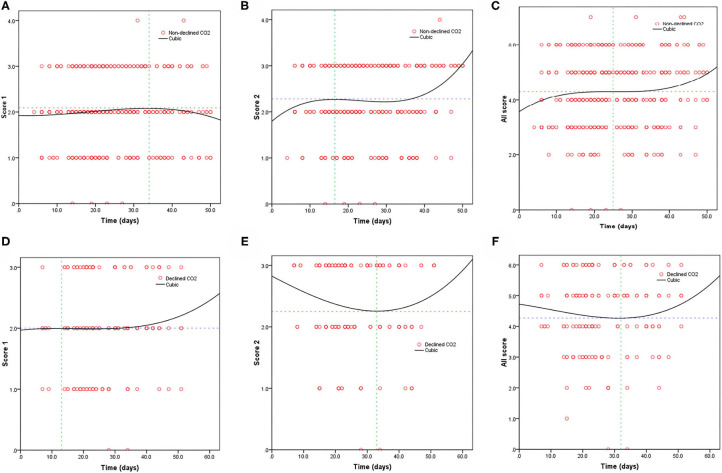
Fitting curves of patients with COVID-19 divided by declined/non-declined levels of CO_2_ based on CT score. Dynamic changes in patients with **(A)** CT score 1 and non-declined CO_2_; **(B)** CT score 2 and non-declined CO_2_; **(C)** total CT score and non-declined CO_2_; **(D)** CT score 1 and declined CO_2_; **(E)** CT score 2 and declined CO_2_; and **(F)** total CT score and declined CO_2_.

Table 3 shows the mortality risk of COVID-19 patients with decreased and non-decreased CO_2_ levels. Both unadjusted and adjusted Cox regression analyses showed that decreased CO_2_ levels were associated, with poor prognosis compared to non-decreased CO_2_ levels. After adjustment for age, history of cardiovascular disease, WBC, PLT, oxygen support, and lymphocyte count, the odds ratio for decreased CO_2_ levels in COVID-19 patients were 4.754 [95% confidence interval (CI): 1.380–16.370, *P* = 0.013]. The hazard ratio for decreased CO_2_ levels in COVID-19 patients was 8.710 (95% CI: 2.773–27.365, *P* < 0.001), and 4.754 (95% CI: 1.380–16.370, *P* = 0.013) after adjustment. Furthermore, the Kaplan-Meier curves illustrated that patients with decreased CO_2_ levels faced higher mortality risks (Figure 1). With the fitted curves, though, in Figure 2A, the curves of patients with non-decreased CO_2_ levels showed a slight downward trend, the CO_2_ levels of most patients were increased (Figures 2B–F).

**Table 3:** The hazards ratio and odds ratio associated with decreased CO_2_ of patients with COVID-19 mortality/severity.

	**Group**	**COX regression analysis**			**Logistic regression analysis**		
		**HRs**	**95 % CI**	***P*-value**	**ORs**	**95 % CI**	***P*-value**
Univariate analysis	Non-declined	Ref			Ref		
	Declined	8.710	2.773–27.365	<0.001	1.213	0.617–2.384	0.575
Multivariate Analysis^*^	Non-declined	Ref			Ref		
	Declined	4.754	1.380–16.370	0.013	0.950	0.431–2.094	0.900

* *Adjust for Age, History of cardiovascular disease, Erythrocyte count, Hemoglobin, Leucocyte count, Platelet count, Lymphocyte count, Oxygen support*.

## Discussion

In this latest outbreak of pneumonia due to COVID-19, patients initially presented with fever with or without respiratory symptoms, although various degrees of pulmonary abnormalities developed later in all patients (1, 12). Furthermore, Tian et al. reported the early phase of the lung pathology of COVID-19 pneumonia in a lung cancer excision, which exhibited edema, proteinaceous exudate, focal reactive hyperplasia of pneumocytes with patchy inflammatory cellular infiltration, and multinucleated giant cells. However, hyaline membranes were not prominent (13). A report demonstrated that the rate of critical illnesses among COVID-19 patients was ~26%, and critically ill patients had 61.5% mortality (12, 14). In another study from Wuhan, the 28-day mortality of COVID-19 patients who received mechanical ventilation (MV) was 81%, and patients with the acute respiratory distress syndrome (ARDS) had a mortality rate of nearly 50% (14, 15). Thus, it is undisputed that the lungs and airways are the target organs of this coronavirus infection.

In this study, we first proposed the correlation of patient prognosis with decreased CO_2_ levels. According to the adjusted logistic regression, Cox regression analyses, and Kaplan-Meier curves, decreased CO_2_ levels influenced the mortality of patients with COVID-19, but not disease severity. Furthermore, this influence on mortality did not differ by sex. However, decreased CO_2_ levels in patients with comorbidity of cardiovascular disease or older age indicated poorer prognosis. Moreover, blood coagulation parameters, such as prothrombin time, international normalized ratio, active partial thromboplastin time, and D-dimer level, showed significant differences between COVID-19 patients with decreased and non-decreased CO_2_ levels; however, decreased CO_2_ levels showed no significant differences in other laboratory parameters.

The measurement of the CO_2_ level is vital in airway management. Capnography is an effective method for the early detection of impaired airway function to identify early respiratory depression and airway disorders (16–18). For example, capnography presented results range 5–240 s earlier than dose pulse oximetry, and in many cases with sedation-induced apnea, doctors at the bedside did not recognize the apnea, whereas capnography could identify it (19, 20). Furthermore, capnography reduces serious complications by early diagnosis (16) and plays a critical role in detecting the CO_2_ level of COVID-19 patients, in whom the target organs are the lungs and airways.

Elevated CO_2_ levels and hypoxemia were associated with a poor prognosis in COVID-19 patients. For example, in the study conducted by Nuckton et al. (21), elevated CO_2_ level likely reflected ARDS severity and an increased dead space fraction. Similarly, Yang et al. reported that most COVID-19 patients usually develop severe pneumonia and are at a high risk factor of ARDS (22). Furthermore, Buchner et al. directly identified that patients with more severe CO_2_ retention might have a poor prognosis (23). Thus, most pneumonia patients with high CO_2_ levels had poor prognosis.

In our study cohort, we found that decreased CO_2_ levels increased mortality but had no significant effect on the disease severity. According to previous studies, the causes of decreased CO_2_ levels are as follows: shortness of breath, reduction of pulmonary perfusion and increased alveolar dead space, and MV hyperventilation (11, 24, 25). Because most COVID-19 patients require various forms of oxygen support, among other treatments, we thought that clinicians should focus their attention on MV hyperventilation (26), which is an effective and practical measure to improve patients' survival. Furthermore, according to the fitted curves, compared with pneumonia patients with non-decreased CO_2_ levels, the other study groups' trend showed an initial decrease and subsequent increase in CO_2_ levels. This indicates that the oxygen flow was adjusted to meet the patients' requirements to treat pneumonia and prevent a decrease in the CO_2_ levels due to hyperventilation.

This study has several limitations. Because the Leishenshan hospital was rapidly built as a designated hospital for COVID-19, it was difficult to share laboratory testing data with other hospitals. Thus, the data may be biased. For example, according to our study, there was no correlation between decreased CO_2_ levels and illness severity in COVID-19 patients. Furthermore, the mechanism of how oxygen support influences CO_2_ levels and thus affects patients' prognoses requires laboratory verification. However, this study makes a significant scientific contribution by providing evidence indicating that clinicians should pay attention to decreased CO_2_ levels in pneumonia patients with COVID-19, and so to prevent hypocapnia and maintain homeostasis.

In this study, we demonstrated that decreased CO_2_ levels increased the mortality risk of COVID-19 patients, but showed no significant impact on the severity of pneumonia. Furthermore, our study serves as evidence for clinicians to pay greater attention to the oxygen flow in COVID-19 patients who receive oxygen support to avoid treatment-related injuries. With these changes, the complications of COVID-19 can be further reduced, thereby improving the prognosis of COVID-19 patients with pneumonia.

## Data Availability Statement

The original contributions presented in the study are included in the article/supplementary material, further inquiries can be directed to the corresponding authors.

## Author Contributions

SC, JH, YH, ML, and WL undertook the research. SW and QL performed the analyses and interpretation of data. DH, JL, and RG wrote the main manuscript text and prepared figures. ZL, LG, and XW revised the article critically for important intellectual content and final approval of the version to be submitted. All authors contributed to the design of the study, writing of the manuscript, reviewed, and approved the manuscript.

## Conflict of Interest

The authors declare that the research was conducted in the absence of any commercial or financial relationships that could be construed as a potential conflict of interest.

## Abbreviations

COVID-19: coronavirus disease
CO_2_: carbon dioxide
CI: confidence interval
SARS-CoV-2: severe acute respiratory syndrome coronavirus 2
CT: computed tomography
GGO: ground-glass opacities
SD: standard deviation
IQR: median and interquartile range
WBC: white blood cell
PLT: platelet
MV: mechanical ventilation
ARDS: acute respiratory distress syndrome.
